# Assessment of Anxiety in Patients With Epilepsy: A Literature Review

**DOI:** 10.3389/fneur.2022.836321

**Published:** 2022-04-25

**Authors:** Raphael Rauh, Andreas Schulze-Bonhage, Birgitta Metternich

**Affiliations:** Epilepsy Center, University Medical Center, University of Freiburg, Freiburg, Germany

**Keywords:** (epilepsy-specific) anxiety, (epilepsy-specific) fear, psychiatric comorbidity, assessment, epilepsy, questionnaire

## Abstract

**Objective:**

Approximately 20% of people with epilepsy (PWE) suffer from anxiety. These fears are quite diverse and may manifest periictally or interictally, be part of the seizure's semiology, or an expression of reactive psychological distress from seizures themselves. Our review addresses the question of what screening tools are used in clinical care and epileptological research to capture the complexity of epilepsy-specific anxieties.

**Method:**

On 2021/11/11, we entered a search string in PubMed that covered our research interest as completely as possible. We also screened the bibliographies of our findings and followed PubMed's recommendations. From the assessments we found in the included studies, we extracted domains that represent the range of manifestations of anxiety, in order to compare the tools and to discuss to what extent they are suitable for assessing epilepsy-specific anxieties.

**Results:**

We screened 1,621 abstracts. In total, we identified 24 different anxiety assessments. In addition to the psychiatric assessments in use, we found 7 tools that were designed to assess epilepsy-specific anxieties. The latter focus on different aspects of epilepsy-specific anxieties. In some cases, the conceptual frameworks are not sufficiently transparent or divergent.

**Conclusion:**

Because a diagnosis of epilepsy can result in, or seizures may appear as, anxiety, it is important to better understand this psychological burden and address it therapeutically, if necessary. There is a need for screening tools that integrate specific points of a variety of assessments, so as to cover the broad range of epilepsy-specific fears. None of the assessments we found meets this integrative perspective. At the same time, the appropriate design of such a required tool presupposes a conceptual framework of what should be considered as epilepsy-specific anxiety.

## Introduction

People with epilepsy (PWE) suffer from anxiety more frequently than the general population and patients affected by other chronic diseases (1). The recognition of the association of psychiatric issues and distress with epilepsy has a long history (2), and the need for assessing psychiatric comorbidities for an adequate therapy has received increasing awareness (3). Whereas psychiatric disorders and depression in particular are considered as relevant comorbidities (4), anxiety has been under-researched so far. Recent studies, however, suggest that in people with epilepsy, anxiety is at least as frequent as depression and dysphoria (5). In recent years the different forms of anxiety in PWE and their pathophysiological and clinical appearance have been debated. These do not only differ in their temporal relation to seizures, but also in terms of their subjective quality and behavioral consequences (5, 6). Thus, anxiety does not appear to be a uniform comorbidity of epilepsy but rather encompass a spectrum of manifestations. This spectrum also represents different pathogenetic mechanisms, including preictal prodromes possibly indicative of proictal alterations in excitability, direct neurobiological mechanisms related to the involvement of brain structures involved in emotional perception and regulation, early ictal anxiety reflecting a loss of control, and interictal anxiety, e.g., in expectation of further seizures, and social stigma manifesting as phobic behavior (7, 8).

Available screening tools have been discussed [e.g., (9, 10)]. Presently, in both epileptological research and clinical care of PWE, a number of questionnaires with different aims have been used, including scales that explicitly or implicitly address the multiple aspects of anxiety, e.g., assessments of quality of life (11–13), social functioning (14), health locus of control (15), or psychological flexibility (16).

In this paper, we have limited our analysis solely to those instruments that are explicitly dedicated to the task of assessing (epilepsy-specific) anxiety in adults. We analyze how anxiety has been assessed in patients with epilepsy to date, which aspects are covered by standardized self-report questionnaires for the general population or instruments specifically developed for people with epilepsy. We also wished to investigate if there are aspects which have been reported qualitatively, but may not have been sufficiently included in standardized assessments so far. For this purpose, a standardized literature search was carried out to identify studies performing an assessment of anxiety in people with epilepsy. Inventories are described and compared concerning their coverage of different conceptual aspects of anxiety. Results are analyzed and discussed with regard to the appropriateness and completeness of assessment of types of anxiety by the respective questionnaires.

## Method

To represent the state of research on the topic comprehensively, we entered the following search terms into PubMed/Medline:

epilepsy OR seizure AND (anxiety OR ictal fear OR “psychiatric comorbidity”)NOT covid NOT “adverse effects” NOT “side effects”

We selected “humans” as an additional filter, excluded studies for people under 18 years old, and only searched for studies with an available abstract.

This strategy was chosen to obtain broad overview of anxiety research in human epileptology. To ensure coverage of the complexity of anxiety's phenomenology, we entered inclusively “ictal fear” (without quotation marks, so that “fear” is respected as a search term on its own) and “psychiatric comorbidity” in addition to the term “anxiety.”

We did not specifically assess the question to which extent Covid-19 might be associated with anxiety in patients with epilepsy; neither did we wish to study “adverse effects” or “side effects” of medical treatment that might trigger anxieties. Nevertheless, publications were considered insofar as they also discussed issues of medical treatment as epilepsy-specific anxiety (see below).

**Figure 1:**
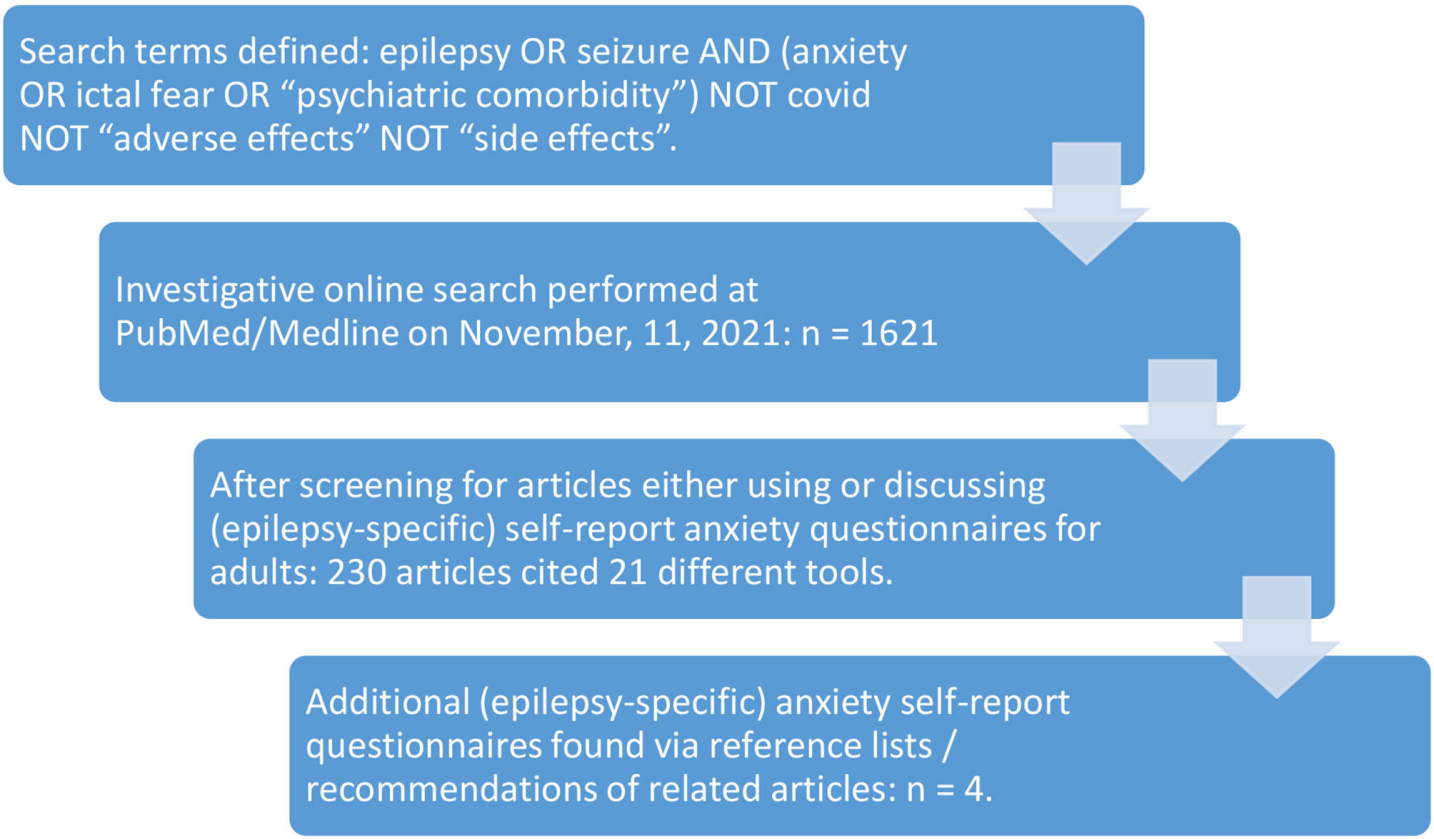
Illustration of the search strategy and selection process.

The search was entered on November 11, 2021, and resulted in 1621 findings. No language limits were selected. Abstracts were screened for relevance, and we checked which screening tools were applied. Both studies applying general anxiety screening tools, as well as studies that designed assessments for epilepsy-specific anxieties, were included, as were papers that discussed forms of anxiety in PWE conceptually (not listed in Figure 1). We also considered the literature lists of screened articles and suggestions for similar articles on PubMed/Medline.

### Selection of Domains

We compared the questionnaires and extracted different domains for the purpose of comparison. Here, we had in mind the main anxiety forms classified by ICD-10 and DSM-5 and the corresponding symptomatology, and the epilepsy-specific forms of anxiety that are discussed in epileptological research. Such domains have been used, e.g., in the Hamilton Anxiety Rating Scale that subsumes the mentioned symptomatology under concise attributes (17), and are further elaborated here and complemented with domains essential to the coverage of epilepsy-specific phenomena.

An analysis of the questionnaires with regard to conceptual categories covered resulted in the following domains:

1. Anxious mood and worries as symptoms that occur in generalized anxiety disorder.
2. Emotional expressions of anxiety (or its opposite), like feelings of tension, irritability, fatigability, restlessness, or weakness.
3. Fear of concrete things, situations, people, including reactive avoidance behavior, reflecting specific phobias, such as social phobia or agoraphobia.
4. Extreme anxiety and panic representing the acute distress as it appears in panic disorders.
5. Somatic symptoms, including muscular and sensory, cardiovascular, gastrointestinal, respiratory, genitourinary symptoms and sleep-related problems.
6. Autonomic symptoms such as blushing or sweating.
7. Cognitive and mental symptoms like compulsive thoughts or hypervigilance.
8. Specific and reactive behavior as a domain represents the behavioral consequences for an individual suffering from anxiety, e.g., having trouble to relax due to inner tension or compulsive thoughts.
9. Specific fears related to anti-epileptic medication (AEM) and its side-effects.
10. Other manifestations of anxiety.

## Results

**Figure 2:**
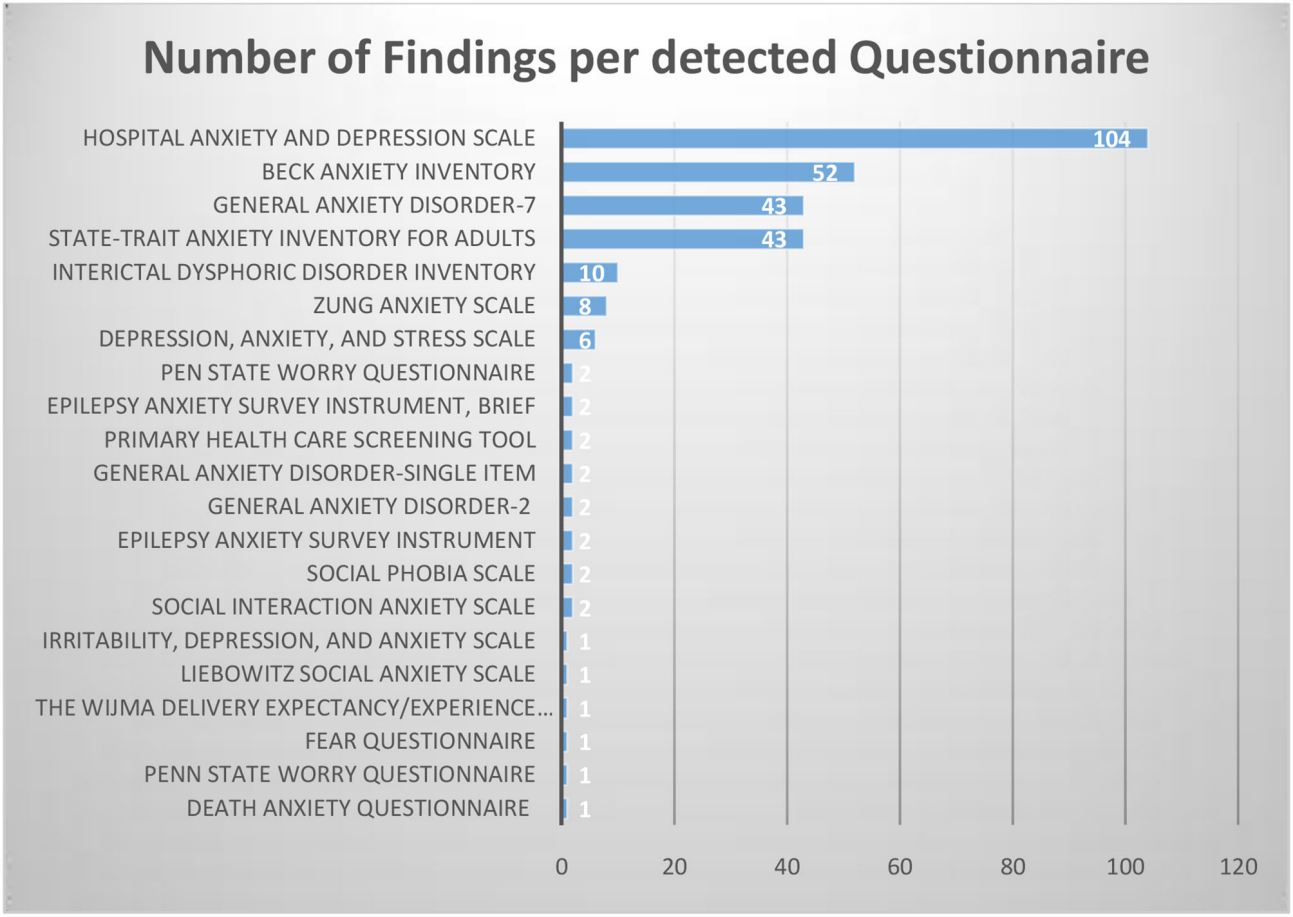
Number of detections per questionnaire.

We identified a total of 21 different self-report questionnaires used for adult PWE in both research and clinical care. Figure 2 shows that the search strategy resulted in common psychiatric questionnaires on anxiety, but also in questionnaires focusing focusing on specific topics like, for example, the Death Anxiety Questionnaire by Otoom et al. (18) or inventories of Interictal Dysphoric Disorder.

We identified four further questionnaires by reading the reference lists of relevant publications and following the recommendation lists at PubMed. Except the Social Phobia Inventory (19), these are methodologically quite different attempts to capture epilepsy-specific anxiety: Bhalla-Gharagozli Fear in Epilepsy Questionnaire (20), Disease-related Fear Scale (21), Fear of Seizures Scale (modified version) (22). Table 1 gives an overview of the questionnaires discussed in our review.

**Table 1:** Summarizes the questionnaires in alphabetic order and categorizes them according to basic features: Number of items, survey period, survey scale, time taken to administer, domains covered.

**Name of assessment/questionnaire**	**Items**	**Time taken to administer (minutes)**	**Survey Period**	**Survey Scale**	**Domains assessed**	**Validation for PWE reported**	**Reliability for PWE reported**	**specific for PWE**	**Comments**
Beck anxiety inventory (BAI)	21	5	Past month	4-point Likert scale	Emotional expression; fear of things, situations, people; extreme anxiety/panic; somatic symptoms; autonomic symptoms; specific/reactive behavior	No	No	No	Puts focus on somatic (sensory and muscular) and autonomic anxiety expressions
Bhalla-gharagozli fear in epilepsy questionnaire (BG-FEQ)	6	2	Not specified	Dichotomous: yes/no	Fear of things, situations, people; somatic symptoms; AEDs	Yes	Yes (the alpha coefficeint was 92,8)	Yes	Epilepsy-specific items, focuses on epilepsy's and medication's consequences with unique items
Depression, anxiety, and stress scale (DASS-21)	21 (7)	9 (3)	Last week	4-point Likert scale	Anxious mood/worries; fear of things, situations, people; extreme anxiety/panic; somatic symptoms; autonomic symptoms	No	No	No	Focus on somatic and autonomic symptoms
Death anxiety questionnaire (DEAQ)	20	10	Not specified	5-point Likert scale	Fear of things, situations, people; others	No	No	Yes	Epilepsy-specific items considering death anxiety in PWE
Disease-related fear scale (D-RFS)	30	10	Not specified	4-point Likert scale	Anxious mood/worries; fear of things, situations, people; somatic symptoms; AEDs; others	Yes	Yes (cronbach alpha:.921); test-retest: no value provided)	Yes	Epilepsy-specific items covering fears of seizure consequences and fear of disease's long-term consequences
Epilepsy anxiety survey instrument (EASI)	18	10	Past 2 weeks	4-point Likert scale	Anxious mood/worries; emotional expression; fear of things, situations, people; extreme anxiety/panic; cognitive/mental impairment; specific/reactive behavior	Yes	Yes (cronbach alpha:.94; test-retest: 0.77) (*p* < .000,5)	Yes	Epilepsy-specific items covering mainly interictal forms of anxiety
Epilepsy anxiety survey instrument, brief (brEASI)	8	5	Past 2 weeks	4-point Likert scale	Anxious mood/worries; emotional expression; fear of things, situations, people; extreme anxiety/panic; cognitive/mental impairment; specific/reactive behavior	Yes	Yes (cronbach alpha:.94; test-retest: 0.79) (*p* <0.0005)	Yes	Epilepsy-specific items covering mainly interictal forms of anxiety
Fear questionnaire (FQ)	24	15	Not specified	Mixed (see description)	Emotional expression; fear of things, situations, people; cognitive/mental impairment	No	No	No	Valuable tool for assessing especially specific phobias and their intensity
Fear of seizure scale (FSS)	15	10	Not specified	8-point Likert scale	Emotional expression; fear of things, situations, people; cognitive/mental impairment specific/reactive behavior; AEM; others	No	No	Yes	Older screening tool for assessing the fear of seizures construct
General anxiety disorder 7 (GAD-7)	7	5	Past 2 weeks	4-point Likert scale	Anxious mood/worries; emotional expression; fear of things, situations, people; specific/reactive behavior	Yes	Yes	No	Short and concise tool to screen for (interictal) GAD
General anxiety disorder 2 (GAD-2)	2	12	Past 2 weeks	4-point Likert scale	Anxious mood/worries; emotional expression	No	No	No	Value for clinical or academic use questionable
General anxiety disorder single item (GAD-SI)	1	1	Past 2 weeks	4-point Likert scale	Specific/reactive behavior	No	No	No	Value for clinical or academic use questionable
Hospital anxiety and depression scale (HADS-A)	7	5	Past week	4-point Likert scale	Anxious mood/worries; emotional expression; extreme anxiety/panic; somatic symptoms; specific/reactive behavior	Yes	Yes	No	Short and concise questionnaire whose items cover a wide range of anxiety's phenomenology
Irritability, depression, and anxiety scale (IDA)	18 (5)	10 (5)	Not specified	4-point Likert scale	Anxious mood/worries; emotional expression; autonomic symptoms; somatic symptoms; specific/reactive behavior	No	No	No	Valuable tool for assessing overlapping symptoms with specific relevance in PWE
Interictal dysphoric disorder Inventory (IDDI)	19 (6)	15 (5)	Not specified	Mixed (see description)	Anxious mood/worries; extreme anxiety/panic	Open	Open	Open	Epilepsy-specific questionnaire, focuses on timely association to the ictus
Liebowitz social anxiety scale (LSAS)	24	15	Past week	4-point Likert scale	Fear of things, situations, people	No	No	No	Valuable tool for assessing (epileptic) social phobia plus resulting avoidance behavior; items limited to this form of anxiety only
Penn state worry questionnaire (PSWQ)	16	10	Not specified	5-point Likert scale	Anxious mood/worries; fear of things, situations, people; cognitive/mental impairment; specific/reactive behavior	No	No	No	valuable tool for assessing a central symptom of (epileptic) generalized anxiety disorder
Primary health care screening tool (PHCST)	10 (5)	5	Past month	4-point Likert scale	Anxious mood/worries; emotional expression; somatic symptoms; cognitive/mental impairment	Yes	Yes (cronbach alpha: 0.57)	Questionable	Tool for assessing general symptoms of anxiety and depression in PWE
Social interaction anxiety scale (SIAS)	20	10	Not specified	5-point Likert scale	Anxious mood/worries; emotional expression; fear of things, situations, people; cognitive/mental impairment; specific/reactive behavior	No	No	No	Valuable tool for assessing (epileptic) social phobia, items limited to this form of anxiety only
Social phobia inventory (SPIN)	17	10	Past week	5-point Likert scale	Fear of things, situations, people; somatic symptoms; autonomic symptoms	No	No	No	Valuable tool for assessing (epileptic) social phobia, items limited to this form of anxiety only
Social phobia scale (SPS)	20	10	Not specified	5-point Likert scale	Anxious mood/worries; fear of things, situations, people; extreme anxiety/panic; somatic symptoms; autonomic symptoms; cognitive/mental impairment; specific/reactive behavior	No	No	No	Valuable tool for assessing (epileptic) social phobia; items limited to this form of anxiety only
State-trait anxiety inventory for adults (STAI-S)	20	10	Present moment	4-point Likert scale	Anxious mood/worries; emotional expression; cognitive/mental impairment; specific/reactive behavior	Yes	Yes	No	Important screening-tool to assess state-anxiety, not of special value for PWE
State-trait anxiety inventory for adults (STAI-T)	20	10	In general	4-point Likert scale	Anxious mood/worries; emotional expression; cognitive/mental impairment	Yes	Yes	No	Important screening-tool to assess trate-anxiety, not of special value for PWE
Wijma delivery expactancy/experience questionnaire (W-DEQ A & B)	33	15	Before (A) and after (B) birth	6-point Likert scale	Emotional expression; fear of things, situations, people; somatic symptoms; cognitive/mental impairment; specific/reactive behavior	No	No	No	Valuable tool for assessing fear of childbirth (of specific relevance in PWE)
Zung anxiety scale (ZAS)	20	10	Past few days	4-point Likert scale	Anxious mood/worries; emotional expression; somatic symptoms; autonomic symptoms	No	No	No	Valuable screening-tool that covers many anxiety domains

*The table shows which publications on anxiety assessments report validity and reliability in epilepsy patients. If reported, statistics are provided (for references see description in our results). A short evaluation can be found in the comments section*.

Not all of the assessments found could be included in the structure of our review. We found older studies using questionnaires on anxiety in the context of epileptology research and care that are no longer in use today, including the Morbid Anxiety Inventory (23). Furthermore, one study combined items from the General Health Questionnaire 5 and the Crown-Crisp Experiential Index to assess anxiety (24). Burton and Labar (25) compiled their own questionnaire to assess the emotional status after right vs. left lobectomy, with 1 item including anxiety (“Feeling nervous and anxious”). Finally, we found a study in (26), in which the Emotional Thermometer-7 is in use, a visual analog scale, for associating anxiety and quality of life in PWE.

### Identified Questionnaires Applied in the Assessment of Anxiety in People With Epilepsy

In the following section, questionnaires covering aspects of anxiety in their assessment are discussed. We arranged our findings systematically in three main categories: comprehensive assessment of anxiety (covering anxiety symptoms in general), focused assessment of anxiety (covering specific types of anxiety), and assessment of epilepsy-specific forms of anxiety.

### Comprehensive Assessment of Anxiety

#### Beck Anxiety Inventory

The BAI consists of 21 questions. The items are one-word descriptions of symptoms considered to address subjective feelings (“nervous”, “unsteady”, “shaky/unsteady”, “scared”, “faint/lightheaded”). Specific fears of dying and of losing control are covered by 2 items. Two items belong to the extreme anxiety/panic domain (“fear of worst happening”, “terrified or afraid”). Eight items focus on somatic expressions of anxiety: 2 muscular, 4 sensory, 1 cardiovascular, 1 gastrointestinal, 2 respiratory. Three items ask for autonomic symptoms. Finally, 1 item addresses the behavioral level (“unable to relax”).

Thus, the BAI is a screening tool covering symptoms especially related to generalized anxiety disorders and panic attacks. Out of the epilepsy-specific fears, it covers ictal and interictal panic disorders well and interictal GAD. Other anxiety forms, such as anticipatory anxiety disorder or epileptic social phobia, are not covered.

#### Depression, Anxiety, and Stress Scale

The DASS-21 consists of a total of 21 items, 7 on depression, 7 on anxiety, and 7 on stress. The 7 explicit anxiety items cover anxious mood, social phobias and panic experience, somatic muscular (hand tremor), cardiovascular (palpitations), respiratory (shortness of breath) and autonomic (dry mouth) symptoms.

The DASS-21 is focused on somatic and autonomic symptoms, as they typically occur during panic attacks. Additionally, 3 of 7 stress items (“I found it hard to wind down”, “I found it difficult to relax”, “I felt that I was using a lot of nervous energy”) address stress symptoms associated with a spectrum of anxiety forms. From the wide range of inter- and periictal fears occurring in PWE, the fear (and stress) items are mainly limited to panic experience and specific phobias including somatic expressions, i.e., epileptic social phobia, ictal fear, and panic disorder. Other epilepsy-specific fears, such as anticipatory anxiety or fears related to medication, are not included.

#### Fear Questionnaire

The FQ consists of three sections. The first section consists of 17 items. The patient is asked to indicate on a scale from 0 (“would not avoid it”) to 8 (“always avoid it”) which situations he would avoid as they cause him anxiety or unpleasant feelings. The first item must be written down by the patient as his individual “main phobia,” which he wishes to be treated. In this section, fears of concrete things, situations and people are assessed, which are also relevant in PWE. For example, the fear of “Hospitals,” “Injections or minor surgery,” “Going alone far from home,” “Thought of injury or illness,” and “Large open spaces” are relevant fears in PWE.

In the second section, the patient is asked to indicate the present state of his actual phobic symptoms on a scale from 0 (“no phobias present”) to 8 (“very severely disturbing/disabling”).

The third section again consists of 6 items directly asking about psychological problems on the emotional level: 1. “miserable or depressed”; 2. “irritable or angry”; 3. “tense or panicky”. Item 4 asks for disturbing thoughts, item 5 for depersonalization experiences, and, finally, item 6 asks for “other feelings” and can be answered individually. Again, these items are to be measured in intensity on a scale from 0 (“hardly at all”) to 8 (“very severely troublesome”).

#### Hospital Anxiety and Depression Scale

The HADS is a questionnaire with 14 items, 7 addressing depression, 7 anxiety. The 7 anxiety items cover anxious mood (worrying thoughts), anxious feelings (tense and frightening feelings), panic (sudden feelings of panic), somatic anxiety symptoms (“butterflies” in the stomach) and reactive behavior (sit at ease/relax vs. restlessness).

The 7 items cover a wide range of manifestations of anxiety relevant to PWE, like interictal GAD and panic disorder. There is no explicit reference to seizures, as in all anxiety questionnaires that were not designed for PWE. Thus, crucial epilepsy-specific forms of anxiety remain unconsidered.

The HADS is discussed as a valid and reliable screening tool in PWE (27). For its methodological evaluation in Temporal Lobe Epilepsy patients, see Zingano (28).

#### Irritability, Depression, and Anxiety Scale

The IDA consists of 18 items. 5 items ask about anxiety, 5 about depression, 4 items about outward directed irritability, and 4 items about inward directed irritability. The 5 anxiety items cover 4 of our domains, including anxious mood and worries, emotional expression of fear, somatic symptoms, and specific or reactive behavior. As only 5 items ask explicitly for anxiety, not all anxiety domains can be covered. In addition to anxiety, the IDA also covers symptoms of irritability and depression, which play a crucial role in PWE.

#### State-Trait Anxiety Inventory for Adults

The STAI is a self-evaluation scale with 20 items probing for state and trait anxiety. The state-anxiety items ask about acute anxiety at the present moment, while the trait-anxiety items ask about how the respondent feels in general.

The state-anxiety items focus on the current feeling dimension. Thus, they ask partly about positive and partly about negative feelings. A total of 15 items ask about the following states with the antecedent phrase “I feel”: nervous, steady, calm, secure, strained, at ease, upset, satisfied, frightened, comfortable, content, pleasant, indecisive, confused, self-confident. Another 5 items have the description “I am” (tense, relaxed, presently worrying over possible misfortunes, jittery, worried) as a prefix. Some of these emotion categories overlap with our seventh domain (cognitive symptoms) (confused, indecisive, self-confident).

The state-anxiety items, on the other hand, have only 8 items with the prefix “I feel” (pleasant, nervous and restless, satisfied with myself, like a failure, rested, that difficulties are piling up so that I cannot overcome them, secure, inadequate). The 12 remaining items ask about general and more persisting personality traits and beliefs (e.g., “I take disappointments so keenly that I can't put them out of my mind,” “I get into a state of tension or turmoil as I think over my recent concerns and interests”).

The STAI is not designed to capture different forms of anxiety. Rather, it is designed to distinguish whether anxiety is a persistent personality trait or a transient emotional experience. This question is also relevant with regard to epilepsy-specific anxieties.

The STAI is discussed as a valid and reliable tool for the use in PWE (29).

#### Zung Anxiety Scale

The ZAS is a questionnaire with 20 items on anxiety that addresses acute anxiety. The ZAS covers a wide range of anxiety expressions, from basic anxious mood, anxious feelings, muscular and sensory-somatic, gastrointestinal, cardiovascular, respiratory, genitourinary, autonomic symptoms, and sleep problems.

Although the symptoms are clearly associated with anxiety and cover several categories, it does not allow to differentiate specific types of anxiety. However, the variety of somatic anxiety symptoms and responses have particular relevance in PWE, for example, anxiety during prodromal or early ictal phases typical for some types of TLE.

### Focused Assessment of Anxiety

#### Generalized Anxiety Disorder-7

GAD-7 is a 7-item self-report questionnaire. The instrument is used to survey Generalized Anxiety Disorder and is the only questionnaire that has been validated in several languages for the use in PWE (see discussion). This international standardization may be one reason why the ILAE promotes it as a screening tool. One item falls into the domain of anxious mood and worry, 2 items address anxious feelings, 1 item addresses compulsive thoughts, and finally 3 items fall into the category of specific reactive behaviors (trouble relaxing, restlessness, becoming annoyed/irritable).

GAD-7 can be used for the identification of (interictal) GAD in PWE. However, other interictal and periictal aspects of anxiety are not covered by this assessment.

#### General Anxiety Disorder-2 and General Anxiety Disorder-Single Item

The GAD-2 is an ultra-short screening tool designed to detect GAD as a distillation of the GAD-7 with 2 items remaining. The 2 remaining items “Not being able to stop or control worrying” and “feeling nervous, anxious or on edge” are shortened to a single item in the GAD-SI: “Trouble Relaxing.” These short and ultra-short screening tools for use in PWE are discussed by Micoulaud-Franchi et al. (12) and Munger Clary et al. (30). Obviously 2 items cannot cover the entire spectrum of anxieties relevant for PWE.

#### Liebowitz Social Anxiety Scale

The LSAS is a questionnaire designed to assess the severity of social phobias. The items describe concrete situations that are expressions of social phobias, such as “telephoning in public,” “going to a party,” “working while being observed,” or “meeting strangers.” At the same time, the extent to which these specific fears result in avoidance behavior is assessed. The LSAS is specific to social phobias and the avoidance behavior that may result from them. Other fears that are equally relevant to PWE are not covered by this questionnaire.

#### Penn State Worry Questionnaire

The PSWQ consists of 16 items. The focus are worries in different facets and contexts. These worries are a crucial symptom of Generalized Anxiety Disorder. In addition to general unspecific worries, sorrows related to projects or tasks are also covered by some items. Furthermore, an obsessive character of the worries, which can manifest itself as a bothering uncontrollable cognition, is also covered.

The PSWQ is particularly useful for assessing generalized anxiety disorder, from which PWE may also suffer. In addition, epilepsy-related worries can bother PWE in many different regards, for example, disease-related memory deterioration, side effects of AEMs, or performing daily routines like shopping or going to work. Other forms of anxiety that may play a significant role in the lives of PWE, such as panic or social phobias, are not assessed by the PSWQ.

#### Social Interaction Anxiety Scale

The SIAS consists of 20 items. It focuses on social anxiety, in particular on its expression in social interactions. It asks about the respondent's psychological and physical wellbeing when socializing with strangers, making eye contact, talking to authority figures, or attractive persons of the opposite sex.

The SIAS is designed to assess the extent to which social anxiety manifests itself in interactions and makes them more difficult. These aspects also play a role in PWE [see also (31)]. Detecting them can be important in some cases, such as when targeting them through cognitive behavioral therapies. However, this questionnaire does not cover a variety of other epilepsy-typical fears.

#### Social Phobia Inventory

The SPIN consists of 17 items and is designed to assess social phobia. It addresses in detail situations in which social anxiety may arise (dealing with authority figures, parties, strangers, embarrassment in front of others, public speaking, etc.) and the resulting avoidance behavior. In addition, somatic and autonomic reactions that arise during social interactions are assessed, such as heart palpitations, blushing, and sweating.

SPIN is designed for the assessment of social phobia, which is of importance also for PWE. However, other epilepsy-specific fears, such as periictal fears, are not considered.

#### Social Phobia Scale

The SPS consists of 20 items. The focus is on fears that arise in social contexts. It covers many fear expressions, from anxious mood to fears caused by situations in interactions with people, to panic expressions. Somatic and autonomic symptoms are also addressed, as are mental aspects and finally reactive behaviors.

The SPS places a focus on social phobias, while also including somatic and autonomic responses. Social phobias may be epilepsy-specific; nevertheless, other epilepsy-specific, inter- and periictal fears are not addressed.

#### The Wijma Delivery Expectancy Questionnaire—Version A and B

The W-DEQ is a questionnaire designed to assess fear of childbirth. Version A asks about expectations of labor and birth, and version B asks about how labor and birth actually went. The items in the two versions are identical in content. For clarity, we discuss here only version A. The 33 items are divided into 6 categories. It asks first about the expected outcome as a whole (with “fantastic” and “frightful” as items), second, it asks generally about feelings during labor and delivery (with, for example, “lonely,” “afraid,” “desolate,” “tense,” “glad,” “abandoned,” “relaxed” as items), third, it asks for expected feelings during labor and birth (with, for example, “panic,” “hopelessness,” and “pain” as items), fourth, it asks what is expected to happen during labor and birth (e.g., “I will totally lose control over my body”), fifth, it addresses the feelings at the very moment of delivery (with e.g., “natural” or “dangerous” as items), and finally sixth, it is asked whether negative thoughts have determined the past concerning labor and birth (like the death of the child).

These items refer to a very specific fear, which makes integration into our domains rather tricky. However, fears as emotional expressions, fear of things and situations, extreme panic, somatic symptoms, cognitive and mental impairment, and specific behavior are covered by the items.

Fear of childbirth may be of great importance to PWE for several reasons. It may be directed more broadly to the inheritance of the disorder or to a negative impact of anti-seizure medication on the child, but also to the possibility of experiencing a seizure during delivery.

### Assessment of Epilepsy-Specific Forms of Anxiety

#### Bhalla-Gharagozli Fear in Epilepsy Questionnaire

Gharagozli et al. (20) discuss fears related to epilepsy and further the psychometric properties of the BG-FEQ. The authors identify 6 epilepsy-specific fears: fear of brain tumor, of premature death, of more frequent/severe seizures over time, fear of suffocation, fear of addiction to medication and fear of adverse effects of long-term intake of anti-seizure medication (AEMs).

The BQ-FEQ addresses two epilepsy-specific fear aspects not covered by other assessments, the fear of addiction and the fear of a brain tumor. However, it is not completely clear on the basis of which methodological considerations the items were selected.

#### Death Anxiety Questionnaire

The DEAQ was developed in a study by Otoom et al. (18). The authors hypothesize that death anxiety plays a particular role in PWE. Twelve items can be most easily assigned to specific fears: dying because of epilepsy, the thought of bereaved relatives after one's own death, fear when medication runs out, fear when the death of other PWE is reported, fear of a painful, sudden, lonely death or death during sleep, fear due to an epilepsy surgery procedure, fear of visiting other PWE in the hospital, sitting beside a dying person, attending a funeral or a corpse washing, or simply the fear of hearing about death. Difficult to assign are the items, “I wish that death was a curable disease” and “I wish that people would not use the word ‘death'.”

The authors state that the above items were assembled from previous studies and adapted for PWE. The validation of the tested items is considered, referring to a Master's thesis discussing death anxiety in cardiac patients, yet not clarified. In the authors opinion, the internal consistency was high (alpha = 0.94) [cf. (18), p. 143].

Inquiring about fears of death as reported by PWE is relevant. For some items, however, it remains open what is specifically meant by fear of death. A theoretical discussion of what subjective character this fear can take, and on the basis of which beliefs it is generated is needed. Due to its specification on death anxiety, the DEAQ does not assess other epilepsy-specific forms of anxiety, such as an epileptic panic attack or epileptic social phobia.

#### Disease-Related Fear Scale

Shamsalinia et al. (21) designed and psychometrically evaluated a disease-related fear scale for PWE. Thirty items are included, divided into fear of the consequences of seizures and fear of long-term damage from the disease. Two items capture a basic anxious mood, a total of 20 items capture concrete fears: the fear of having a seizure during flirting/sex, that the family will lose faith in the cure of the disease, being injured during a seizure, social discrimination, worsening of the disease, brain damage, or even fear of compassion. In addition, 3 somatic fear expressions are assessed: fear of choking, of incontinence during a seizure, and fear of seizures due to insomnia. Five items refer to antiepileptic medication, and 1 item refers to the inheritance of the disease to one's own children.

The authors consider the D-RFS a valid and reliable assessment for PWE [(21), p. 5].

The assessment emerged from interviews with PWE and covers individual medical histories and the individual complexity and heterogeneity of epilepsy-specific fears. The authors themselves point out that the study should be replicated in other cultural contexts. The interviews from which the items were generated were conducted with 14 PWE, a relatively small sample. A larger population in a different cultural context may alter the composition of items.

#### Epilepsy Anxiety Survey Instrument and Brief Epilepsy Anxiety Survey Instrument

The EASI claims to be the first epilepsy-specific validated anxiety screening tool (32). It consists of 18 (long version) or 8 items (brief version). In 9 (EASI) and 6 (brEASI) items, respectively, the reference to epilepsy is not mentioned. In 2 (EASI), respectively, 1 (brEASI) item, the reference to seizures is explicitly excluded (“getting terrified out of the blue, unrelated to my seizures' and 'sudden feeling of panic, unrelated to my seizures”). The methodological reasoning behind this is that periictal fears are part of epilepsy. The authors do not consider these to be fears, as they are pathophysiologically determined as being part of a seizure.

The EASI asks about basic anxious mood, 1 item independent of epilepsy, 1 item related to epilepsy in terms of fear of the next seizure, and the impact on the social environment. A total of 6 items address specific fears and phobias with avoidance behavior. Three items address panic fears. Four items address cognitive and mental aspects of anxiety. Finally, 3 items assess behavioral manifestations.

In the brEASI, all categories of EASI are covered, with a reduced number of items per category.

The items of the EASI were distilled from interviews with PWE and expert opinions. The experts were asked to what extent common anxiety screening tool items (from BAI, GAD-7, and HADS) could be confounded by “aspects of epilepsy, seizures, or AEDs” (p. 2070). However, open questions remain as to which anxiety entities are considered as epilepsy-specific. Also, the methodological premise of separating a possible anxiety semiology of a seizure from epilepsy-specific anxiety necessarily leads to gaps in assessing clinically relevant forms of anxiety in PWE—especially periictal manifestations.

#### Fear of Seizures Scale

The FSS was originally designed by Mittan (33) and revised by Goldstein et al. (22). We only discuss the latter version (the items are the same). It consists of 15 items. It is designed to assess epilepsy-specific fears, namely fear of seizures. Answering patients are first asked about their acute fears, that are addressed by the items, and second about the probability, a respective seizure-related event might happen to them. Thus, the items mainly presume effects and consequences of seizures (e.g., emotional disorders, brain damage, death, a shorten life-span, mental deterioration, tongue swallowing, suffocation), their exacerbation (e.g., more medication needed) and their preconditions (e.g., brain tumors), including reactive avoidance behavior (e.g., physical exertion or exposure to flashing light/loud noise).

The items point to crucial domains of concrete epilepsy-specific fears that are related to seizures. Other symptoms of common psychiatric anxieties, which also play a central role in PWE, such as generalized anxiety disorders or social phobia, are not assessed.

#### Interictal Dysphoric Disorder Inventory

The IDDI covers 8 main symptoms, which are grouped into 3 main categories: labile depressive symptoms (depressed mood, anergia, pain, and insomnia), labile affective symptoms (fear, anxiety), and supposedly ‘specific' symptoms (paroxysmal irritability and euphoric moods) [(34), p. 82]. In the appendix, 6 questions assess the temporal relationship of symptoms, the frequency and duration of dysphoric symptoms, and their relationship to seizures and AEDs. It asks whether dysphoria (only) emerges in the context of seizures, and if so, in what temporal sequence seizures and dysphoria occur, and how long each lasts.

Two main items on “Fear/Panic” (“Do you experience feelings of fear or feel panicky from time to time?”) and “Anxiety” (“Do you have frequent worries, feelings of oppression, agitation, or anxiety from time to time?”) require a dichotomous (“yes” or “no”) response.

Even if the disease entity IDD has been questioned, and it is also unclear whether this syndrome can be identified as epilepsy-specific [e.g., (35, 36)], all symptoms assessed can have relevance for PWE. The IDDI specifically addresses the time of occurrence of anxiety symptoms in relation to a seizure. The 2 main anxiety items cover important epilepsy-specific anxiety experiences, such as an panic attack or interictal GAD; others, however, are missing, as they are not considered to be component of the construct of IDD.

#### Primary Health Care Screening Tool

The PHCST consists of 10 items for assessment of depression and anxiety in PWE. According to the authors (37, 38) explanation, 4 items exclusively refer to anxiety (“thinking about too many things or thinking too much”, “feeling anxious”, “difficulty in concentrating” (in our opinion this item represents a typical depressive symptom, but we follow the authors conception), “experience of increased heartbeat for the past 1 month”), 1 item refers simultaneously to anxiety and depression (“disturbed sleep”). The anxiety items play a significant role in PWE. They cover some typical expressions of anxiety in general. More specific forms of anxiety are not covered by this tool.

The reliability and validity of the tool is discussed in the respective original paper (37).

**Figure 3:**
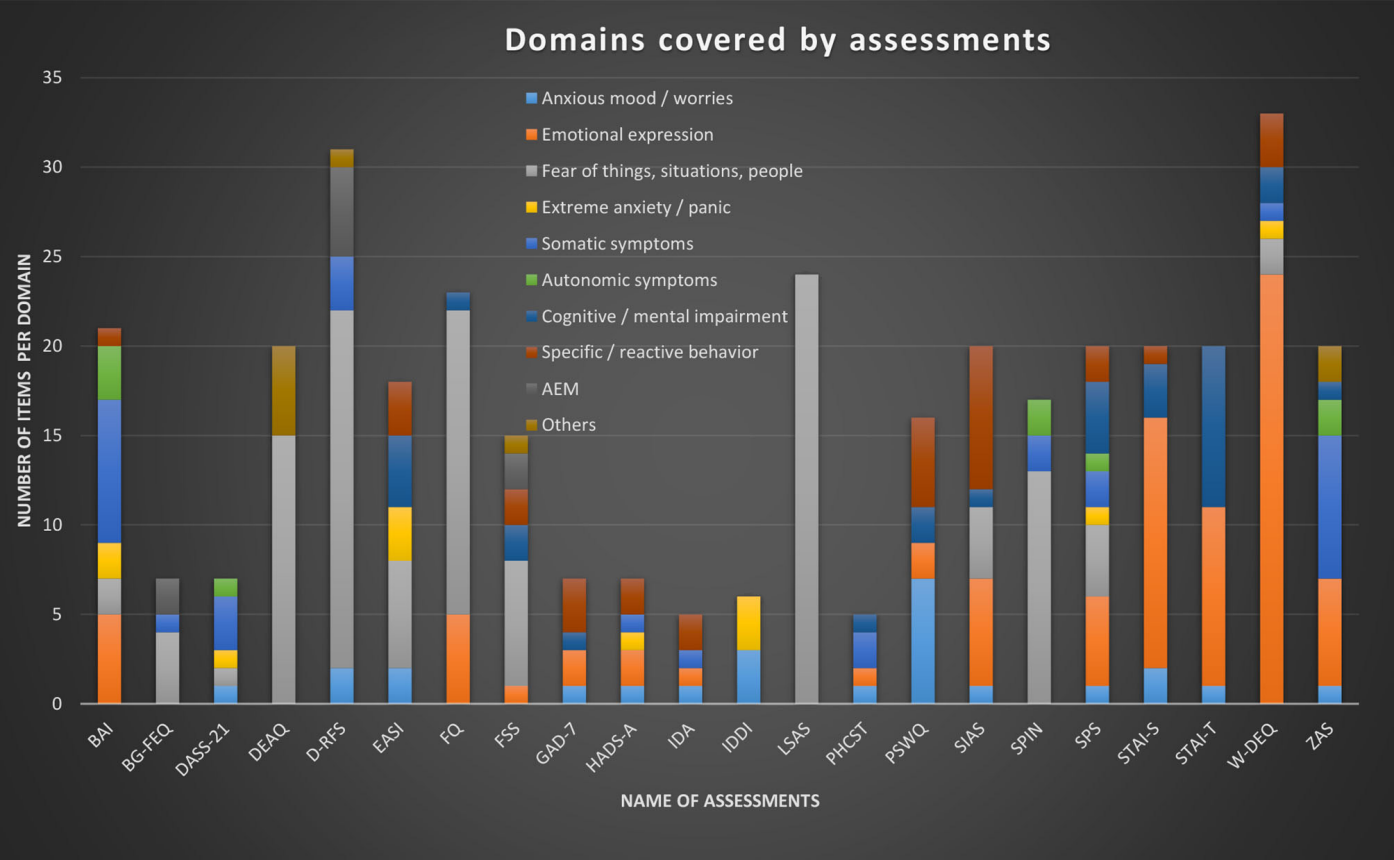
Summary of our findings illustrating and comparing 22 different self-report questionnaires that are in use for the assessment of anxiety in PWE. It illustrates the number of items per questionnaire and their allocation to the domains we have extracted. We have not included the short versions of GAD-7 and EASI (GAD-2, GAD-SI, and brEASI) in this illustration.

Figure 3 summarizes our results in one illustration. The domains can be distinguished by different colors, which allows to compare the focus and range of the different assessments. The height of the column corresponds to the number of items; colors represent different domains, and the distribution of colors in relation to others represents the balance between domains addressed.

## Discussion

The results show that a variety of questionnaires is in use to assess anxiety in PWE for different purposes. We want to highlight two main aspects. First, epileptological research is interested to better understand the spectrum of forms of anxiety in PWE, and its manifestation in, and interrelation to, different types of epilepsy and demographic data. Questionnaires are used to advance research in understanding the relationship between the prevalence of specific fears in PWE and the mechanisms involved in their genesis as well as the correspondence to specific types of epilepsy.

Second, practitioners are interested in supporting their patients with best available care, anxiety being a debilitating experience with negative impact on the quality of life. Questionnaires are then of interest to detect the occurrence of anxiety and to include this knowledge in the treatment. A better theoretical understanding of the epilepsy-specific forms will likely lead to better health care, and vice versa. We identified:

- Questionnaires designed to capture anxieties comprehensively or with regard to specific forms of anxiety, yet, without addressing epilepsy-specific aspects.
- epilepsy-specific questionnaires that target aspects which are presumed to be specific forms of anxiety in PWE and aim to map them accordingly.

The usefulness of the identified assessments in PWE critically depends on the conceptualized epilepsy-specific forms of anxiety (39), which we will briefly discuss in the following. An official standardized classification of epilepsy-specific fears is not yet available. It is still an open question if psychiatric comorbidities should be considered in the classification of epileptic disorders (40).

### Forms of Anxiety in PWE

A temporal distinction is made between periictal (preictal, ictal, and postictal) and interictal fears (5, 6, 41). Hingray et al. (5) and Ertan et al. (6) also formulate open questions for assessing these entities.

Periictal anxieties mainly include early ictal-aware perception of fear (“fear auras” according to the old terminology (42) and “ictal fears,” “subjective ictal anxiety,” or “ictal panic” of temporal lobe epilepsy with involvement of the amygdala and other limbic structures (43). They can be misdiagnosed as panic attacks due to their similar clinical appearance (44). These neurobiological forms of fears have no particular nomenclature. Postictal anxiety can arise from phases with impaired awareness with retrograde amnesia of the patient's ictal behavior.

Interictal fears (5) include the “anticipatory anxiety of epileptic seizures.” This form of anxiety describes a strong fear directed at the expected occurrence of further seizures. “Seizure phobia” is a particularly disturbing form of this anticipatory anxiety, which is accompanied by intensified thoughts and avoidance behaviors regarding certain circumstances, places, situations where seizures have already occurred or might occur. “Epileptic social phobia” means the intense fear of being watched by others during a seizure. This phobia may also refer to particular aspects of the seizure type, including loss of consciousness, motor phenomena, postural instability or autonomic signs with highly stigmatizing potential like drooling or enuresis. A resulting avoidance of social interactions is a particularly debilitating aspect, as it may persist over time and render the patient prone to loneliness and social isolation. “Epileptic panic disorder” is according to (5) a specific panic disorder that occurs in association with agoraphobia, an entity that is not described by other researchers, as far as we know. In our opinion this supposedly interictal form of anxiety could be an expression of ictal fear, thus, could be a part of the seizure semiology. The term “fear of seizures” (45) is quite extensive and refers to fears that follow from assumed preconditions or consequences of seizures, but also to the fear of losing control during a seizure or the unpredictability of the next seizure (anticipatory anxiety, see above).

Last but not least, iatrogenic anxieties play a role for different therapeutic approaches. Thus, they may be related to anticipated or experienced adverse effects of medication (5, 7), and a fear of non-adherence and its possible consequences in terms of seizure exacerbation. Treatment-related anxiety is a matter, particularly when brain surgery is offered to control seizures. A possible transient increase in anxiety or *de novo* anxiety is reported after surgical interventions (46).

These entities provide some orientation, but are neither strictly defined nor a sufficient or exhaustive reflection of epilepsy-specific fears, which are described and experienced as individually as the personal disease histories.

### Assessments for Epilepsy-Specific Forms of Anxiety

Our search identified seven epilepsy-specific assessments targeting anxiety.

The BQ-FEQ consists of six items that query epilepsy-specific fears. It is the only questionnaire addressing fear of addiction to the prescribed antiepileptic medication. Addressing this phenomenon and actively discussing it with patients may be of relevance for the establishment of a trustful atmosphere and therapeutic adherence. The five other items are nearly identical with the items in the FSS (see below).

The DEAQ by Otoom et al. (18) strives for a more accurate understanding of fear of death in PWE. The authors do not explicitly delve into which death anxieties should be considered. Thus, real seizure-related risks of dying during the course of a seizure due to a fall, in status epilepticus, or as a result of autonomic dysfunction in SUDEP can fuel death fears. However, some patients also describe ictal fear as something like a subjective premonition or experience of death that does not refer to seizure-related risks but is rather a particular ictal experience. Further, items are somewhat heterogeneous as they also address fear of a surgical procedure or of medication running out. Some items seem to be suited to special cultural contexts. The item of the fear of corpse washing, for example, is only meaningfully assessed in a context where this practice still has relevance.

The D-RFS consists of 30 items that were derived deductively from the literature and inductively from patient interviews. This addresses and captures a particularly comprehensive range of issues. In particular, fear of both the immediate consequences of seizures and of long-term sequelae are captured. The range of fears thus covered is very specific, with most items relating to public space or social interactions. The D-RFS items refer to social, peer, and family support, sexual intimacy, stigma, and discrimination, but also more subtle and irrational fears, such as social isolation due to the contagiousness of the disease or the fear of being harassed by the pity of the social environment. In contrast, other epilepsy-specific fears of a subjective nature, including anxiety as part of the seizure semiology, are not included in this assessment.

The EASI and its abbreviated version emerged from patient interviews and expert opinions. Experts were asked to what extent items of common psychiatric anxiety assessments could be confounded by genuinely epileptic symptomatology. That is, the authors did not consider anxiety that is part of seizure semiology as epilepsy-specific anxiety. This is an *a priori* methodological decision distinguishing this approach from other assessments, but it leads to gaps in assessing periictal forms of anxiety which patients experience and report as anxiety. Neglecting or excluding this subjective experience as such, just because the underlying pathophysiology is epileptic, is certainly debatable.

The EASI scores particularly well with respect to interictal fears, which are comprehensively covered by the items. The EASI is the only epilepsy-specific assessment that limits the survey period, referring to the past 2 weeks only. This decision has the advantage of being able to assess recent experiences that make a recall bias unlikely. On the other hand, it is possible that potentially existing interictal fears are not captured because the period considered is too short. The International League Against Epilepsy's homepage offers a free download of the EASI/brEASI as an alternative tool to the GAD-7 for assessing comorbid and interictal anxieties in PWE.

The FSS is an older instrument to assess epilepsy-specific fears of seizures that include subjective, often unfounded attitudes or expectations concerning seizures. They are frequently experienced by PWE and might subconsciously alter cognitive or behavioral attitudes. It is important to address them early after the first diagnosis of epilepsy since it may be reassuring for PWE to have them reflected in medical knowledge and facts. Further, they may have a negative influence on emotional and behavioral adjustment.

The IDDI assumes a specific syndrome, which also includes anxiety symptoms. The time course of dysphoric mood plays a crucial role. A particular advantage of this questionnaire is that it does not only categorize the presence of experienced pain, insomnia, anxiety and panic, etc., but additionally captures the intensity of these experiences and also the impact on the quality of life in a semi-quantitative manner. The appendix also allows for further differentiation, including, for example, the temporal relation to seizures. Of note, some researchers doubt that interictal dysphoric disorder is a distinct diagnosis and specific to PWE (for references see description in our results).

The PHCSTs obviously screens for symptoms of anxiety and depression. But as a matter of fact, the items are not specific for PWE, but assess typical anxiety and depression symptoms that can be found in the general population, too.

From these descriptions, it becomes evident that these tools vary in their theoretical and practical usefulness. The BQ-FEQ, D-RFS and the DEAQ, FSS, and the IDDI analyze the spectrum of anxiety in PWE. The EASI/brEASI is the most practical tool to assess interictal anxiety in PWE. High scores in any of the mentioned tools may trigger considerations of therapeutical consequences. Many epilepsy-specific anxieties can be eased by just naming and addressing them in a protected environment with the physician. For a discussion of pharmacological and (underresearched) psychotherapeutical treatment of anxiety in PWE see (5, 47).

### Common Psychiatric Anxiety Assessments

The common psychiatric anxiety assessments found in our literature search differ significantly. The STAI's questions whether state- or trait-anxiety is present are also relevant for anxiety in PWE. An important question in this context is whether patients with an anxious personality would benefit from other therapies than patients whose fears turn out to be related more closely to epilepsy. Studies are furthermore needed to investigate to which degree anxiety traits reflect the chronic course of the disease. Questionnaires such as the SIAS, SPIN, SPS, LSAS, or GAD-7 specifically ask about defined psychiatric disease entities that have a counterpart in PWE. However, they are inappropriate for generating a picture of the many different forms that anxiety can take in PWE. Shortening the GAD-7 to GAD-2 or GAD-SI again results in a highly incomplete coverage of manifestations of anxiety in PWE.

The use of the GAD-7 as a screening tool for comorbid anxiety in PWE has been promoted by the International League Against Epilepsy. A free version can be downloaded at the ILAE's homepage. It has been validated for the use in PWE and is available in different translations: Chinese (48), French (49), Indonesian (50), Korean (51), Russian (52). The availability in multiple languages allows to perform cross-cultural comparisons in different populations. Of note, the cutoff of the GAD-7 is lower in PWE compared to the general population [for different interpretations, see (53)]. In general, different cutoff points may serve to draw attention to specific comorbidities and address these, rather than to establish the diagnosis of a disorder of its own as is a typical use in the general population, which may favorably use a higher cutoff score.

The W-DEQ asks for fear of childbirth, which often is relevant in PWE. The PSWQ asks for milder but nonetheless disturbing forms of anxiety, including compulsive thoughts. The FQ addresses mainly specific phobias and leaves space for differentiation therein, insofar as the patient can state his individual main phobia. The ZAS and BAI questionnaires are remarkable in that they include a range of sensory, muscular-somatic, and autonomic symptoms. Of note, some infrequently applied questionnaires such as the DASS-21 or IDA cover a wide range of the domains we have differentiated and ask for symptoms relevant to PWE, including irritability, stress, and depression.

Overall, it is clear that the forms of anxiety that PWE may suffer are particular forms of anxiety insofar as they are related to experiencing seizures. These fears are at most, if at all, only indirectly captured by general anxiety assessments.

## Limitations

The present review's results are restricted by the applied search string. A broader search, not excluding terms such as “covid” or “adverse effects” would have resulted in a larger count of initial hits, and perhaps further relevant questionnaires would have emerged. However, such a strategy would also have resulted in a much larger number of abstracts to screen, which would have been beyond the scope of our resources. Another restriction is the limitation to self-report questionnaires only due to the same reasons. Fiest et al. (10) discuss other forms of assessments beyond self-report questionnaires, such as semi-structured interviews. Another limitation of our narrative review is that we did not discuss the methodological robustness of the different assessments. In contrast, Wang et al. (54) provide a systematic review regarding validated anxiety questionnaires for PWE. But they do not mention any tool for epilepsy-specific anxieties. In contrast, our narrative review covers a larger number of questionnaires that are actually used in epileptological research and clinical practice. Further, it discusses and compares for the first time seven questionnaires that take on the task of assessing epilepsy-specific forms of anxiety.

## Conclusion

The psychological burden of a diagnosis of and living with epilepsy is pronounced and complex. While affective disorders have received more in-depth attention in epileptology, anxiety—despite its high prevalence—has received relatively little attention.

Often patients initially feel overwhelmed and left alone with the diagnosis of epilepsy. Early consideration of the psychological distress that a patient goes through may help to alleviate this burden, either by simply addressing it directly or, if necessary, through appropriate therapeutic measures. A routine assessment of epilepsy-specific fears with appropriate instruments may be helpful in this context.

Overall, the spectrum of symptoms and signs of anxiety in PWE identified in this review is noteworthy and reflects the multitude of phenomenological aspects of anxiety present in PWE. On the other hand, none of the questionnaires reported here covers all relevant epilepsy-specific fears. Instead, the assessments are focused on particular aspects and domains. There is still a lack of validated screening tools that cover the wide range of anxiety's phenomenology in PWE. This calls for future developments of more comprehensive assessment strategies covering the variety of epilepsy-related anxieties, which can be used to screen for and thoroughly assess issues relevant to the heterogeneous population of PWE.

## Author Contributions

RR performed the literature research, evaluated the literature, and wrote the manuscript. AS-B designed the study, participated in writing, and the discussion of results. BM assisted with the literature search, performed corrections of the manuscript, and discussed results. All authors contributed to the article and approved the submitted version.

## Funding

We acknowledge support by the Open Access Publication Fund of the University of Freiburg.

## Conflict of Interest

The authors declare that the research was conducted in the absence of any commercial or financial relationships that could be construed as a potential conflict of interest.

## Publisher's Note

All claims expressed in this article are solely those of the authors and do not necessarily represent those of their affiliated organizations, or those of the publisher, the editors and the reviewers. Any product that may be evaluated in this article, or claim that may be made by its manufacturer, is not guaranteed or endorsed by the publisher.
